# Supplementary material for: Human antibodies targeting Zika virus NS1 provide protection against disease in a mouse model
Source: Nat Commun. 2018 Nov 1;9:4560. doi: 10.1038/s41467-018-07008-0 (PMC6212565; doi:10.1038/s41467-018-07008-0)
Supplement: Supplementary file 1 — Supplementary Information [file 41467_2018_7008_MOESM1_ESM.pdf]

Human antibodies targeting Zika virus NS1 provide protection against disease in a mouse model

Bailey et al.

Antibody	Viral Strain	$K_{on}1$ (1/Ms)	$K_{dis}1$ (1/s)	K_D1 (M)	$K_{dis}2$ (1/s)	R^2	$K_{on}2$ (1/Ms)	K_D2 (M)	X^2
AA12	MR766	$1.10x10^{4}$	$6.11x10^{-4}$	$5.54x10^{-8}$	$1.62x10^{-2}$	0.9989	$1.63x10^{5}$	$9.98x10^{-8}$	0.6389
AA12	PRVABC59	$6.03x10^{3}$	$4.59x10^{-4}$	$7.61x10^{-8}$	$2.13x10^{-2}$	0.9986	$1.45x10^{5}$	$1.47x10^{-7}$	0.2677
FC12	MR766	No binding	No binding	No binding	No binding	No binding	No binding	No binding	No binding
FC12	PRVABC59	$7.98x10^{3}$	$5.70x10^{-4}$	$5.33x10^{-8}$	$1.70x10^{-2}$	0.9977	$3.18x10^{5}$	$7.15x10^{-8}$	0.0658
EB9	MR766	$2.24x10^{4}$	$6.52x10^{-4}$	$2.92x10^{-8}$	$9.45x10^{-3}$	0.9992	$1.15x10^{5}$	$8.22x10^{-8}$	1.7281
EB9	PRVABC59	$2.23x10^{4}$	$6.29x10^{-4}$	$2.81x10^{-8}$	$9.93x10^{-3}$	0.9996	$1.36x10^{5}$	$7.31x10^{-8}$	1.0758
GB5	MR766	$2.22x10^{3}$	$7.18x10^{-4}$	$2.71x10^{-7}$	$3.55x10^{-4}$	0.9967	$8.30x10^{4}$	$3.23x10^{-7}$	0.0267
GB5	PRVABC59	$9.69x10^{3}$	$7.45x10^{-4}$	$7.69x10^{-8}$	$1.84x10^{-2}$	0.9985	$1.73x10^{5}$	$1.06x10^{-7}$	0.0756

Supplementary Table 1. Affinity of antibodies determined by surface plasmon resonance. Biolayer interferometry assays were performed with an Octet RED instrument (ForteBio, Inc.) to determine K_D values. Purified recombinant NS1 protein from either MR766 or PRVABC59 viruses was loaded onto a Ni-NTA biosensor and kinetic constants were obtained as follows. K_{on} was determined by exposing sensors to diluted antibody. K_{off} was determined by exposing the sensors bound to antibody to buffer alone. K_D values were calculated as the ratio of K_{off} to K_{on}. As a 2:1 binding model is used, K1 and K2 are defined as the first and second measured antibody interaction with antigen. R^2 and X^2 values measure the variability and goodness of fit and were calculated using ForteBio software and acceptable values are R^2 close to 1 and X^2 under 3.

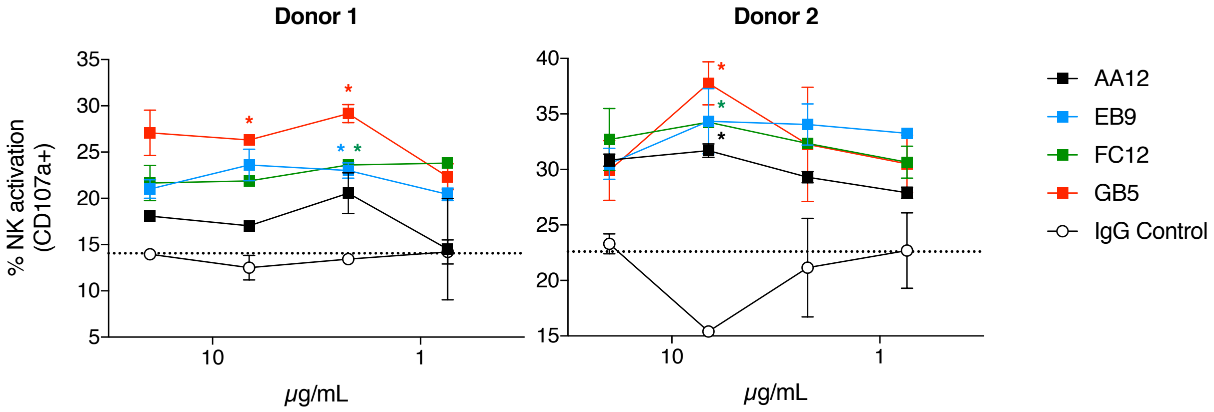

Supplementary Figure 1. Activation of human primary natural killer cells. Vero cells were infected with PRVABC59 at an MOI of 0.5. At 48 hpi, dilutions of mAb (starting at 20 µg per mL), irrelevant mAb or media containing no mAb (negative control) were added to ZIKV-infected Vero cells and incubated for 1 hour at 37ºC. Primary human NK cells from donor **a.** 1 and **b.** 2 were then added (effector to target ratio of 2) and the culture was further incubated at 37ºC for three hours. The cells were then stained with CD56 (FITC) and CD107a (PE). Activation of NK cells (expression of CD107a) were detected using a flow cytometer and analyzed using Flowjo software. Data represent percent of NK cells expressing CD107a and are the mean value of duplicates and the SEM. A multiple t-test and the Holm-Sidak method was performed using GraphPad Prism. Significance (* = $p < 0.05$) is indicated compared to control IgG. Dotted line on the y-axis represent the average value of the negative control group (media with no mAb).

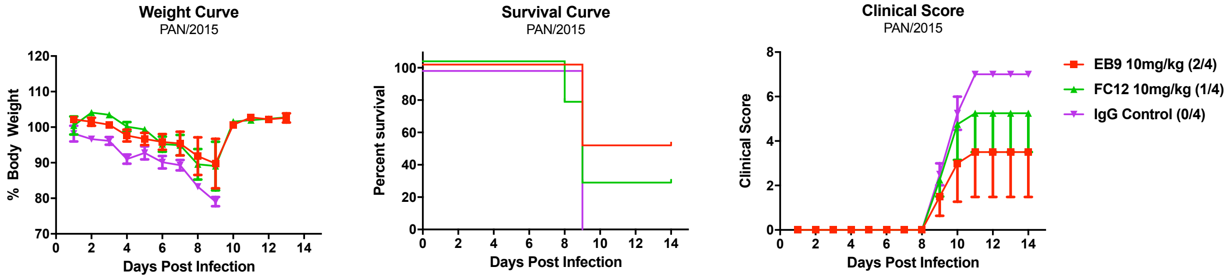

Supplementary Figure 2. Challenge model of NS1-specific antibodies against PAN/2015. Groups of 4 male and female B6.129-Stat2$^{-/-}$ mice were injected IP with 10 mg per kg of EB9, FC12, or IgG control before a challenge with 500 PFU of ZIKV PAN/2015 retro-orbitally. **a, b.** Weight loss was monitored daily and mice that lost 25% of their original weight were sacrificed. **c.** Clinical scoring was conducted using the pre-defined criteria with a maximum possible score of 7: impact on walking (1), unresponsiveness (1), left hind leg paralyzed (1), right hind leg paralyzed (1), left front leg paralyzed (1), and right front leg paralyzed (1). Deceased animals were given a score of 7. The ratios in the figures indicate the number of animals that survived challenge over total number of animals per group. Statistical analyses were performed using the Mantel-Cox and Gehan-Breslow-Wilcoxon tests for the survival curves and a multiple t-test and the Holm-Sidak method for the weight curve and the clinical score. No significant differences between groups were detected in **a, b** and **c**.

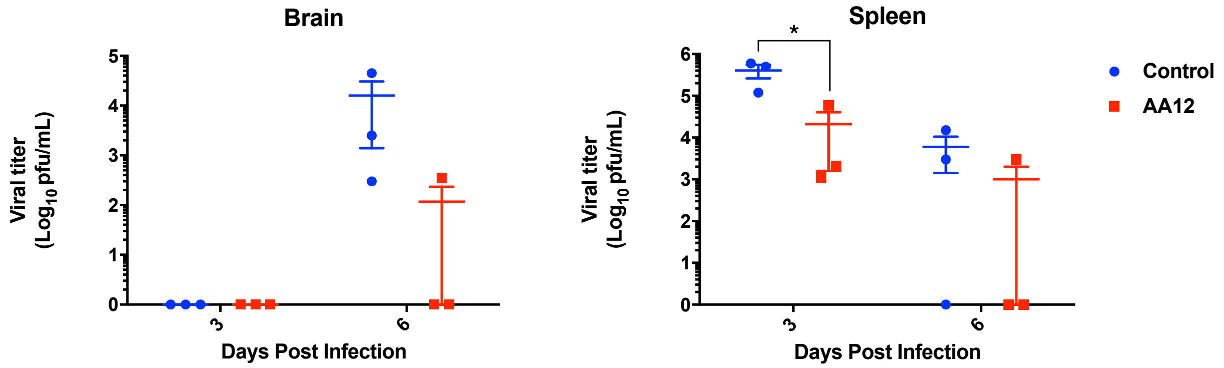

Supplementary Figure 3. Viral burden in brains and spleens of infected mice. Groups of 6 male and female B6.129-Stat2$^{-/-}$ mice were injected IP with 10 mg per kg of AA12 or IgG control before a challenge with 500 PFU of ZIKV PAN/2015 retro-orbitally. **a.** Brains and **b.** spleens were harvested at days 3 and days 6 post infection. Viral titers were determined by plaque assay. Error bars represent SEM. A two-way ANOVA followed by a Holm-Sidak multiple comparison analysis was performed using GraphPad Prism 5 and yielded a significant difference between control and AA12-treated mice in the spleen at 3 dpi.

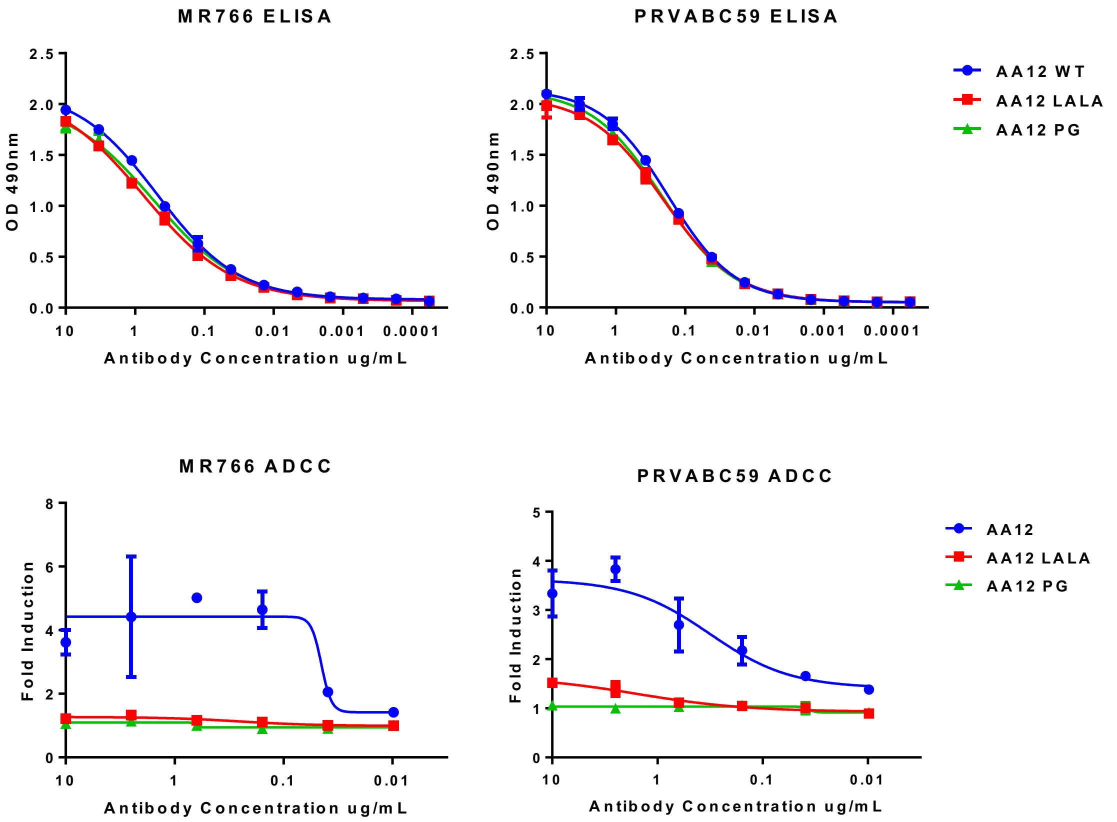

Supplementary Figure 4. LALA and PG Fc-variants of AA12.. a, b. ELISA assays were performed using recombinant NS1 from either MR766 and PRVABC59 viruses to assess the binding activities of mAbs AA12 and AA12 variants. **c, d.** Fc-FcR mediated effector functions were tested on Vero cells infected with MR766 and PRVABC59 Zika viruses. AA12 was able to elicit Fc-FcR mediated effector functions while AA12 variants were not.

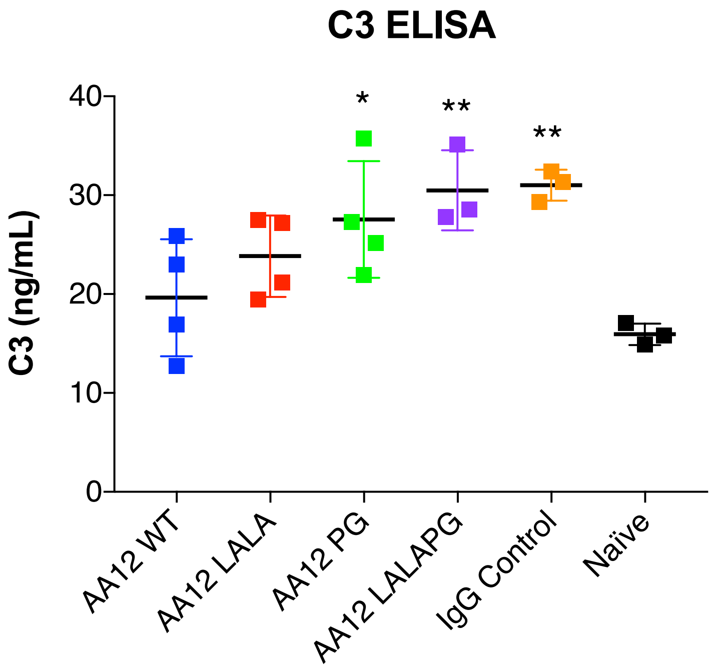

Supplementary Figure 5. Complement levels are raised during acute ZIKV infection. Groups of 3 to 4 male and female B6.129-Stat2$^{-/-}$ mice were injected IP with 10 mg per kg of AA12 wild-type, AA12 LALAPG, AA12 LALA, AA12 PG or IgG control before a challenge with 10 mLD_{50} of ZIKV MR766 intradermally. At day 6 post infection C3 levels were determined by ELISA as per manufacturer's instructions. A one-way ANOVA and Dunnett's multiple comparison tests was performed to determine statistical significance of the mAb-treated groups to the naïve uninfected mice (* = $p < 0.05$ ** = $p < 0.005$).
